# Alcohol and cannabis use severity in relation to compulsive sexual behavior and problematic pornography use in a non-clinical sample: a cross-sectional study

**DOI:** 10.3389/fpsyt.2026.1863547

**Published:** 2026-06-17

**Authors:** Wiktoria Stasica, Alicja Anna Binkowska

**Affiliations:** Faculty of Psychology, Humanitas University, Sosnowiec, Poland

**Keywords:** alcohol, behavioral addiction, cannabis, compulsive sexual behavior, problematic pornography use, thc

## Abstract

**Introduction:**

Compulsive Sexual Behavior Disorder (CSBD) and problematic pornography use (PPU) frequently co-occur with substance use disorders, yet their associations with specific substances in non-clinical populations remain poorly understood. The present study examined associations between alcohol and cannabis use severity and CSBD and PPU symptoms in a non-clinical adult sample.

**Methods:**

Eligible adults were invited to complete an anonymous online survey between May and October 2025. Alcohol and cannabis use severity scores were assessed using AUDIT and CUDIT-R questionnaires, respectively. Participants compulsive sexual behavior disorders (CSBD) and problematic pornography use (PPU) scores were assessed using CSBI-13 and BPS questionnaires. Associations between substance use severity and compulsive sexual behaviors were assessed using linear adjusted regression.

**Results:**

A total of 158 adults (61.4% women) aged 18–60 years were analysed with a mean alcohol and cannabis use severity scores of 16.29 (± 4.53) and 15.49 (± 5.08), respectively among current users, and a mean compulsive sexual behavior disorders of 21.14 (± 7.74), with a mean PPU score of 6.99 (± 2.55). In unadjusted regression models, increases in alcohol and cannabis use severity were associated with increases in CSBD and PPU scores. However, no significant increase of both outcomes was observed for alcohol use severity (CSBD: B = 0.18, 95% CI [-0.13; 0.49], p = .251; PPU: B = 0.04, 95% CI [-0.05; 0.13], p = .381) and cannabis use severity (CSBD: B = 0.09, 95% CI [-0.13; 0.31], p = .409; PPU: B = 0.03, 95% CI [-0.03; 0.09], p = .325) after controlling for age and gender.

**Conclusion:**

Our study showed a positive but non-significant association between alcohol and cannabis use severity with both CSBD and PPU after controlling for age and gender, suggesting the underlying associations may be explained by sociodemographic differences in the study population. However, longitudinal studies and advanced analysis approaches including those that handle zero inflated data or latent dependant variable are needed to validate these findings.

## 1 Introduction

In recent years, growing attention has been devoted to difficulties in controlling sexual behaviors (1). Repetitive and intense sexual behaviors undertaken despite negative consequences across multiple areas of life have become the subject of considerable scientific debate and diagnostic controversy (2–5).

The disorder has been described under various terms, such as paraphilia-related disorder (6), atypical impulsivity disorder (7), and hypersexuality (8). In response to the growing body of research and the need for conceptual clarification, the 11th revision of the International Classification of Diseases (ICD-11) introduced the diagnosis of Compulsive Sexual Behaviour Disorder (CSBD), classifying it within impulse control disorders (9).

According to ICD-11, CSBD is defined as a persistent pattern of failure to control intense, repetitive sexual impulses or urges, manifested in repetitive sexual behaviors, unsuccessful attempts to reduce these behaviors, and their continuation despite negative consequences and little subjective reward or pleasure derived from them. This pattern persists over an extended period of time (e.g., at least 6 months) and is associated with significant clinical distress or impairment in functioning.

CSBD encompasses a broad spectrum of behaviors, such as compulsive masturbation, problematic pornography use, cybersex, casual sexual encounters, and other forms of sexual activity. In many cases, sexual activity serves a function of emotion regulation, including the reduction of tension, anxiety, and feelings of loneliness (10, 11). Over time, this may lead to compulsive engagement in sexual activity and impaired functioning in occupational, relational, and health-related domains (1, 12).

One of the forms belonging to compulsive sexual behaviors is problematic pornography use (PPU) (13). It represents only one of the possible manifestations of CSBD, but requires separate consideration. The rapid expansion of digital technology has significantly influenced patterns of pornography consumption. Accessibility, anonymity, the absence of financial barriers, and the abundance of highly stimulating content have contributed to an increase in problematic pornography use (14, 15). For most individuals, the use of available resources may not constitute a disorder; however, in some cases, it may lead to loss of control over time and frequency of use, unsuccessful attempts to reduce consumption, and continuation of the behavior despite negative consequences (16–18).

Despite the inclusion of compulsive sexual behavior disorder in ICD-11, the literature indicates that these behaviors share important features with behavioral addictions. Kraus et al. (19) suggest that shared neurotransmitter systems may contribute to both compulsive sexual behaviors and substance use disorders, while neuroimaging studies indicate similarities in mechanisms related to craving and attentional processes. Further studies emphasize common neural and behavioral mechanisms associated with anticipatory processing of cues predicting erotic rewards among individuals reporting problematic pornography use, as well as comparable pathophysiological mechanisms, including the compulsive nature of the behavior, difficulty in cessation, and treatment response (13, 20–22). At the same time, the complexity of CSBD - encompassing impulsivity, compulsivity, and emotion dysregulation - has led some researchers to question whether its classification as a behavioral addiction is justified by the available evidence (1, 13, 23, 24).

Alcohol and cannabis are among the most commonly used psychoactive substances worldwide (25, 26). Although occasional use of these substances does not necessarily lead to clinically significant difficulties, higher levels of alcohol and cannabis use severity have been associated with a range of psychological, behavioral, and interpersonal problems. Previous studies indicate that problematic alcohol use is associated with impulsivity, impaired behavioral control, increased risk-taking, and difficulties in emotion regulation (27–29). Alcohol consumption has also been linked to risky sexual behaviors, including earlier sexual initiation, inconsistent condom use, and having multiple sexual partners (30). Similarly, cannabis use has been associated with deficits in inhibitory control, heightened reward sensitivity, difficulties in emotion regulation, and the use of substances as a coping strategy for stress and negative emotions (31, 32). Recent research increasingly emphasizes the role of emotion dysregulation and impulsivity as transdiagnostic vulnerability factors underlying both substance-related and behavioral addictions (33).

These mechanisms may also be relevant to compulsive sexual behavior disorder and problematic pornography use, particularly when sexual activity serves an emotion regulation function. Previous studies suggest that compulsive sexual behaviors frequently co-occur with substance use disorders and other addictive behaviors (8, 34). Difficulties in emotion regulation may contribute to maladaptive coping strategies and increased engagement in compulsive sexual behaviors aimed at reducing distress or regulating negative affect. Similar mechanisms have also been proposed in relation to problematic pornography use and other compulsive sexual behaviors (13).

Despite growing interest in CSBD, relatively few studies have examined its associations with specific substance use patterns in non-clinical populations (35–37). Most available research has focused on broader comorbidity patterns or aggregated substance use measures rather than isolating the effects of individual substances (37, 38). As a result, the distinct contributions of alcohol and cannabis use severity to compulsive sexual behavior and problematic pornography use have received limited empirical attention.

The present study aimed to examine the associations between alcohol and cannabis use severity and compulsive sexual behavior and problematic pornography use in a non-clinical adult sample. Given the limited research on substance-specific patterns in non-clinical populations, alcohol and cannabis use severity were examined separately to explore their potentially differential relationships with sexual behavior outcomes.

## 2 Methods

### 2.1 Study design and participants

The present study employed a cross-sectional design. Eligible participants were invited through media platforms to complete an anonymous online survey between May and October 2025. The survey consisted of providing sociodemographic information as well as information on their health status, alcohol and cannabis use severity, and sexual behavior disorders through validated questionnaires. To be eligible, respondents were required to be aged 18 years or older, living in Poland, and provide a signed consent prior to participation. Participation was voluntary and unpaid.

### 2.2 Measures

#### 2.2.1 Alcohol and cannabis use severity

Alcohol use severity was assessed using the *Alcohol Use Disorders Identification Test* (AUDIT; 39). The instrument comprises 10 items rated on a 5-point scale (0–4), assessing frequency and quantity of alcohol consumption, loss of control, and alcohol-related adverse consequences. The total score is obtained by summing all items, with higher scores indicating a greater risk of problematic alcohol use. In the present study, the scale demonstrated good internal consistency (α = 0.84).

Cannabis use severity was assessed using the *Cannabis Use Disorders Identification Test–Revised* (CUDIT-R; 40). The questionnaire consists of 8 items rated on a 5-point scale (0–4), assessing frequency of use, loss of control, and negative consequences associated with cannabis consumption. The total score represents the sum of all items, with higher scores indicating a greater risk of problematic cannabis use. In the present study, the scale demonstrated good internal consistency (α = 0.85).

#### 2.2.2 Compulsive sexual behavior disorders and problematic pornographic use

Compulsive sexual behavior disorders were assessed using the *Compulsive Sexual Behavior Inventory–13* (CSBI-13; 41). The questionnaire consists of 13 items rated on a 5-point Likert scale ranging from 1 (*never*) to 5 (*very often*). The total score is calculated by summing responses to all items, with higher scores indicating greater severity of compulsive sexual behavior symptoms. In the present study, the scale demonstrated good internal consistency (α = 0.89).

Problematic pornography use was assessed using the *Brief Pornography Screener* (BPS; 42). The instrument consists of 5 items rated on a 3-point Likert scale (0 = *never*, 1 = *sometimes*, 2 = *often*). The total score is computed by summing all items, with higher scores reflecting greater severity of problematic pornography use. In the present study, the scale demonstrated good internal consistency (α = 0.89).

#### 2.2.3 Sociodemographic data

Socio-demographic data were collected using the socio-demographic section included in the questionnaires. They included age, gender, place of residence, education level, relationship status, and relationship duration, as well as mental health status, including diagnosed psychiatric disorders and the use of medications potentially affecting libido.

### 2.3 Ethical considerations

No personal data or IP addresses were collected. The study protocol was approved by the Research Ethics Committee of Humanitas University and preregistered prior to data collection (https://aspredicted.org/cp47-pwjj.pdf).

### 2.4 Statistical analyses

Descriptive statistics were calculated for all study variables. The distribution of alcohol and cannabis use severity variables was examined both in the full sample and among current users. Outliers were defined *a priori* as participants with total scores exceeding ±3 SD from the sample mean on any primary continuous variable (AUDIT, CUDIT-R, CSBI-13, or BPS) and were excluded from all subsequent analyses.

Because gender was included as a predictor variable in regression analyses and as a moderator in interaction models, regression and moderation analyses were restricted to participants identifying as women or men (n = 156). One participant identifying as non-binary was excluded from these analyses.

Primary analyses consisted of unadjusted and adjusted linear regression models examining associations between alcohol and cannabis use severity variables and compulsive sexual behavior (CSBI-13) and problematic pornography use (BPS). To distinguish between substance use status and severity of use, binary indicators of alcohol and cannabis use status (user vs. non-user) were included together with alcohol and cannabis use severity scores (AUDIT and CUDIT-R, respectively). This approach allowed associations with substance use severity to be estimated independently of differences between users and non-users. This approach allowed the coefficients of the substance use severity variables to directly capture the effect of a one-unit increase in severity independently of differences between users and non-users. In unadjusted models, alcohol- and cannabis-related variables were examined separately. Adjusted models included alcohol and cannabis use variables simultaneously together with sociodemographic covariates (age, gender, education level, and relationship status) to estimate adjusted associations. Education level was dummy-coded with higher education (university degree) as the reference category. Robust HC4 standard errors were applied throughout to account for potential heteroscedasticity and high-leverage observations (43).

All analyses were conducted in R (version 4.5.1). Statistical significance was set at α = .05 (two-tailed).

To examine potential gender differences in the association between substance use severity and sexual behavior outcomes, exploratory moderation analyses were conducted including interaction terms (AUDIT × gender and CUDIT-R × gender), adjusting for sociodemographic covariates. All moderation models included the same sociodemographic covariates as the primary regression analyses (age, education level, and relationship status). Robust HC4 standard errors were applied in all moderation analyses.

## 3 Results

### 3.1 Participants’ characteristics

Initially, 160 individuals completed the survey. Two participants were excluded due to outlier scores exceeding ±3 standard deviations from the mean on at least one primary study variable, resulting in a final analytical sample of 158 participants with a mean age of 31.86 years (SD = 8.97, range: 18–60). The majority of participants were women (61.4%, n = 97). Regarding substance use, 68.4% (n = 109) reported current alcohol use and 41.1% (n = 65) reported current cannabis use. In terms of mental health status, 39.2% of participants (n = 62) reported at least one psychiatric or psychological diagnosis, most commonly mood disorders (28.5%) and anxiety disorders (21.5%). Detailed demographic and clinical characteristics of the sample are presented in **Table 1**.

**Table 1:** Demographic characteristics of the study sample (N = 158).

Variable	Category	n	%
Gender	Female	97	61.4
	Male	60	38.0
	Non-binary	1	0.6
Education	Primary/middle education	5	3.2
	Secondary education	33	20.9
	Vocational education	12	7.6
	Higher education	79	50.0
	Currently enrolled in university	29	18.4
Place of residence	Village	11	7.0
	Small town (up to 20,000 inhabitants)	22	13.9
	Medium-sized city (20,000–100,000 inhabitants)	36	22.8
	Large city (over 100,000 inhabitants)	89	56.3
Relationship status	Formal relationship	60	38.0
	Informal relationship	80	50.6
	Polyamorous relationship	1	0.6
	Single	17	10.8
Use of libido-affecting medication	Yes	34	21.5
	No	114	72.2
	I don’t know	10	6.3

### 3.2 Substances use, CSBD and PPU

​​Descriptive statistics for primary study variables are presented in **Table 2**. In the full analytical sample (N = 158), compulsive sexual behavior scores (CSBI-13) were M = 21.14 (SD = 7.74) and problematic pornography use scores (BPS) were M = 6.99 (SD = 2.55). Mean AUDIT score in the full sample was M = 11.24 (SD = 8.44), whereas mean CUDIT-R score was M = 6.37 (SD = 8.31). Among current alcohol users (n = 109), mean AUDIT score was M = 16.29 (SD = 4.53), and among current cannabis users (n = 65), mean CUDIT-R score was M = 15.49 (SD = 5.08).

**Table 2:** Descriptive statistics for primary study variables.

Variable	N	M	SD	Min	Max	α
Compulsive sexual behavior symptoms (CSBI-13)	158	21.14	7.74	13	45	0.89
Problematic pornography use (BPS)	158	6.99	2.55	5	15	0.89
Alcohol use severity - full sample (AUDIT) (full sample)	158	11.24	8.44	0	31	0.84
Alcohol use severity - current users (AUDIT))	109	16.29	4.53	10	31	0.82
Cannabis use severity - full sample (CUDIT-R) (full sample)	158	6.37	8.31	0	31	0.85
Cannabis use severity - current users (CUDIT-R)	65	15.49	5.08	9	31	0.82

For AUDIT and CUDIT-R, full sample statistics include non-users assigned a score of zero; user-only statistics reflect scores among current users only. Descriptive statistics for AUDIT and CUDIT-R are presented both for the full sample and separately for current users.

### 3.3 Association between substance use and CSBD and PPU

Results of unadjusted and adjusted regression models are presented in **Table 3**. In unadjusted models, alcohol use severity was positively associated with both CSBI-13 (B = 0.62, 95% CI [0.39, 0.85], p <.001) and BPS scores (B = 0.16, 95% CI [0.08, 0.24], p <.001). Similar associations were observed for cannabis use severity (CSBI-13: B = 0.31, 95% CI [0.08, 0.54], p = .009; BPS: B = 0.07, 95% CI [0.01, 0.13], p = .018). In adjusted models, none of the substance use variables were significantly associated with either outcome. Gender was significantly associated with BPS scores (B = 2.84, 95% CI [1.88, 3.80], p <.001).,

**Table 3:** Association between substance use severity and compulsive sexual behavior disorders.

	Compulsive sexual behavior disorders				Problematic pornography use			
	Unadjusted		Adjusted		Unadjusted		Adjusted	
	B (95% CI)	p	B (95% CI)	p	B (95% CI)	p	B (95% CI)	p
Alcohol								
Use	-10.51 (-14.37, -6.64)	<.001	[-10.56, 5.94]	.584	-2.67 (-3.95, -1.39)	<.001	-0.57 (-2.84, 1.70)	.623
Use severity	0.62 (0.39, 0.85)	<.001	0.18 (-0.13, 0.49)	.251	0.16 (0.08, 0.24)	<.001	0.04 (-0.05, 0.13)	.381
Cannabis								
Use	-2.11 (-3.87, -0.35)	.020	-1.12 (-4.34, 2.10)	.496	-0.92 (-1.82, -0.01)	.047	-0.22 (-1.08, 0.64)	.617
Use severity	0.31 (0.08, 0.54)	.009	0.09 (-0.13, 0.31)	.409	0.07 (0.01, 0.13)	.018	0.03 (-0.03, 0.09)	.325

Models adjusted for age, gender, education level, and relationship status. B denotes the unstandardized regression coefficient and represents the expected change in the outcome score associated with a one-unit increase in the predictor, holding all other variables constant.

### 3.4 Potential influence of gender

Exploratory moderation analyses examined whether gender moderated the associations between substance use severity and compulsive sexual behavior (CSBI-13) and problematic pornography use (BPS). No interaction effects between gender and alcohol or cannabis use severity reached statistical significance. The largest interaction effect was observed for cannabis use severity and sex in predicting CSBI-13 scores (B = −0.28, 95% CI [−0.58, 0.02], p = .071). All remaining interaction effects were non-significant (**Table 4**).

**Table 4:** Association between substance use severity and compulsive sexual behavior disorders by gender.

	Compulsive sexual behavior disorders (CSBI-13)			Problematic pornography use (BPS)		
	B (95% CI)	p	p interaction	B (95% CI)	p	p interaction
Alcohol use severity			.844			.132
Male	-0.01 [-0.26, 0.24]	.921		-0.05 [-0.13, 0.04]	.276	
Female	0.02 [-0.21, 0.25]	.875		0.03 [-0.03, 0.09]	.308	
Cannabis use severity			.071			.950
Male	-0.00 [-0.25, 0.24]	.983		0.02 [-0.07, 0.11]	.679	
Female	0.27 [0.08, 0.47]	.007		0.02 [-0.03, 0.08]	.434	

## 4 Discussion

The present study examined associations between alcohol and cannabis use severity and symptoms of compulsive sexual behavior (CSBD) and problematic pornography use (PPU) in a non-clinical sample. Our unadjusted regression analysis showed a significant positive association with both CSBD and PPU, for alcohol use severity, whereas cannabis use variables showed a weaker and less consistent associations with these outcomes.

However, most effects were attenuated and became non-significant after controlling for sociodemographic variables.

These findings extend previous research by examining substance-specific associations in a non-clinical population and addressing the limited evidence on whether alcohol and cannabis show differential relationships with CSBD and PPU.The overall pattern of results suggests that associations between substance use severity and compulsive sexual behaviors may be relatively weak and context-dependent in non-clinical populations. This is consistent with prior literature indicating that relationships between CSBD/PPU and substance use severity are often small in magnitude and heterogeneous across studies (37, 44). The attenuation of effects after controlling for sociodemographic variables further suggests that these associations may reflect shared background characteristics rather than direct relationships.

Several mechanisms may account for the observed associations. First, shared vulnerability factors, such as impulsivity and difficulties in emotion regulation, may predispose individuals to both substance use and compulsive sexual behaviors (35, 45). Second, both CSBD and substance use disorders may rely on analogous mechanisms within the reward system, particularly processes described by the incentive sensitization theory (46–48). Additionally, acute use of psychoactive substances may temporarily amplify these tendencies by reducing inhibitory control and increasing reward sensitivity, which may facilitate the escalation of compulsive sexual behaviors (49). However, the weak and inconsistent associations observed in the present study provide only limited support for this explanation. Third, broader lifestyle or sociocultural factors may underlie both substance use severity and sexual behavior patterns. Among these explanations, the shared vulnerability account appears most consistent with the present findings, particularly given the attenuation of associations after controlling for sociodemographic variables, suggesting that broader individual differences may underlie both behaviors.

The present findings do not allow for strong conclusions regarding the extent to which CSBD is closely linked to substance-related addictive processes, particularly in non-clinical populations. Although some theoretical models emphasize shared neurobiological and behavioral mechanisms between CSBD and addictions (19), the small and inconsistent associations observed here suggest that such links may be limited or context-dependent. These findings should be interpreted with caution, especially given the cross-sectional design.

Gender emerged as a robust predictor of problematic pornography use, with men reporting substantially higher levels than women, which is consistent with previous literature (16, 50). However, gender did not significantly moderate the associations between substance use severity and sexual behavior outcomes. Notably, the interaction between cannabis use severity and gender in predicting CSBD approached statistical significance, suggesting a potentially weaker association among men; however, this effect requires replication in larger samples.

From a clinical perspective, the results suggest that substance use may co-occur with compulsive sexual behaviors, but it should not be assumed to play a central or causal role. Screening for substance use in individuals presenting with CSBD symptoms may nevertheless be warranted, particularly given the potential for shared risk factors and comorbidities (34). At the same time, the findings support a more comprehensive assessment approach that considers both sexual behavior patterns and substance use severity, without assuming a direct or uniform relationship between them.

Several limitations should be acknowledged. First, the cross-sectional design precludes conclusions regarding causality or directionality. Second, the use of a convenience sample recruited via social media limits generalizability, particularly given the predominance of women and the overrepresentation of younger, highly educated, and urban participants. These characteristics may have influenced the observed associations and limit the applicability of the findings to populations with different sociodemographic profiles. Furthermore, the predominance of women may have reduced variability in problematic pornography use scores, as men typically report higher levels of PPU, potentially attenuating some of the observed associations. Third, all measures were based on self-report and may be subject to reporting biases (51). Fourth, the study did not assess contextual aspects of substance use, such as use in sexual contexts, which may be particularly relevant for understanding these relationships. Additionally, the relatively small size of the cannabis user subgroup limited statistical power, particularly for detecting smaller effects.

An interesting finding was the opposite direction of associations observed for substance use status and substance use severity in the unadjusted models. While higher alcohol and cannabis use severity were associated with higher CSBD and PPU scores, substance use status showed inverse associations. This may suggest that the mere presence of substance use and the severity of substance-related problems capture different aspects of functioning. In particular, variability in the severity of use among users may be more relevant to compulsive sexual behaviors than user status alone. However, as these associations became non-significant after adjustment for sociodemographic factors, they should be interpreted cautiously and require further investigation in larger samples.

## 5 Conclusion

In this preregistered cross-sectional study conducted in a non-clinical sample, alcohol and cannabis use severity were not significantly associated with compulsive sexual behavior or problematic pornography use after adjustment for sociodemographic factors. These findings suggest that the observed associations may be largely explained by underlying sociodemographic characteristics. The co-occurrence between substance use severity and problematic sexual behaviors may reflect common underlying mechanisms, such as impulsivity and difficulties in emotion regulation, rather than direct effects of specific substances. Future studies should distinguish more clearly between substance use status and severity, incorporate contextual measures of substance use in sexual situations, and use longitudinal designs to clarify directionality.

## Data Availability

The raw data supporting the conclusions of this article will be made available by the authors, without undue reservation.
